# Supplementary material for: Anti-Obesity Effect of Auricularia delicate Involves Intestinal-Microbiota-Mediated Oxidative Stress Regulation in High-Fat-Diet-Fed Mice
Source: Nutrients. 2023 Feb 8;15(4):872. doi: 10.3390/nu15040872 (PMC9962468; doi:10.3390/nu15040872)
Supplement: Supplementary file 1 [file nutrients-15-00872-s001.zip › nutrients-2185937-supplementary.pdf]

Supplementary material

Supplementary Table S1 The effects of ADe on body weight and organ index.

	Week	NCD	NCD+400 mg/kg ADe	HFD	HFD+400 mg/kg ADe	HFD+800 mg/kg ADe	HFD+3 mg/kg Sim
Body weight (g)	0	23.5±0.3	24.5±0.6$^{\#}$	33.4±1$^{\#\#\#}$	32.3±0.9	34.7±1	34.9±1.6
	1	25.1±0.5	23.6±0.4$^{\#}$	34±0.9$^{\#\#\#}$	32.5±1.3	32.2±1.3	34.5±1
	2	26.4±0.4	24.5±0.5$^{\#}$	36.3±1.8$^{\#\#\#}$	33.5±1.6	32.9±1.2	33.4±1.4
	3	26.1±0.4	24.7±0.5$^{\#}$	34.7±0.9$^{\#\#\#}$	35.6±1.4	34.4±1	34.7±1.7
	4	25.8±0.4	24.5±0.3$^{\#}$	39.5±1.1$^{\#\#\#}$	34±1**	36.1±0.8*	37.5±1.3
	5	26±0.4	24.7±0.3$^{\#}$	43.9±1.3$^{\#\#\#}$	36.5±1.2**	37.7±0.8**	37.8±1.3**
	6	26.3±0.5	24.6±0.5$^{\#}$	45.9±1.4$^{\#\#\#}$	39.4±1.5**	38.5±1**	39.5±1.6*
	7	27±0.2	25.8±0.4$^{\#}$	46.4±1.2$^{\#\#\#}$	40.7±1.5*	41.8±1.5*	41.6±1.2*
	8	27.2±0.6	24.7±0.5$^{\#}$	48.2±1.1$^{\#\#\#}$	42.9±0.9**	42.9±1.1**	41.8±2.4*
	9	27.2±0.3	25.4±0.5$^{\#}$	47.9±1.1$^{\#\#\#}$	42.8±1.6*	44.1±1.2*	41.4±2*
	10	27.5±0.5	25.2±0.6$^{\#}$	50.6±1.2$^{\#\#\#}$	44.8±2.2*	45.8±1.5*	45.4±1.9*
Organ index (%)	Spleen	0.239±0.01	0.239±0.01	0.174±0.006$^{\#\#\#}$	0.19±0.001*	0.195±0.006*	0.179±0.005
	Kidney	1.19±0.017	1.255±0.009$^{\#\#}$	0.689±0.022$^{\#\#\#}$	0.852±0.037**	0.834±0.021***	0.825±0.042*
	Heart	0.543±0.018	0.51±0.022	0.384±0.011$^{\#\#\#}$	0.443±0.02*	0.46±0.019**	0.43±0.015*

Data were analyzed using a one-way ANOVA and are expressed as the means ± S.E.M. (n = 6). $^{\#}$ $p < 0.05$, $^{\#\#}$ $p < 0.01$ and $^{\#\#\#}$ $p < 0.001$ versus NCD-fed mice; * $p < 0.05$, ** $p < 0.01$ and *** $p < 0.001$ versus HFD- fed mice.

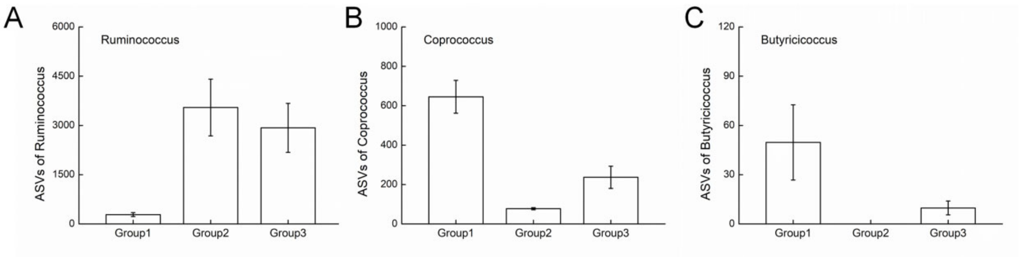

Figure S1 The ASVs number of *Ruminococcus* (**A**), *Coprococcus* (**B**), and *Butyricicoccus* (**C**). Data are presented as the mean ± S.E.M. (n = 3 for NCD-fed mice and HFD-fed mice, and n=4 for ADe-treated HFD-fed mice). Group 1: NCD-fed mice, Group 2: HFD-fed mice, Group 3: ADe-treated HFD-fed mice.
